# Further Insights into the Allan-Herndon-Dudley Syndrome: Clinical and Functional Characterization of a Novel MCT8 Mutation

**DOI:** 10.1371/journal.pone.0139343

**Published:** 2015-10-01

**Authors:** Christine M. Armour, Simone Kersseboom, Grace Yoon, Theo J. Visser

**Affiliations:** 1 Regional Genetics Program, Children’s Hospital of Eastern Ontario, and Children's Hospital of Eastern Ontario Research Institute, University of Ottawa, Ottawa, Canada; 2 Department of Internal Medicine, Erasmus University Medical Center, Rotterdam, The Netherlands; 3 Department of Paediatrics, Divisions of Neurology and Clinical and Metabolic Genetics, Hospital for Sick Children, Toronto, Canada; University Claude Bernard Lyon 1, FRANCE

## Abstract

**Background:**

Mutations in the thyroid hormone (TH) transporter MCT8 have been identified as the cause for Allan-Herndon-Dudley Syndrome (AHDS), characterized by severe psychomotor retardation and altered TH serum levels. Here we report a novel *MCT8* mutation identified in 4 generations of one family, and its functional characterization.

**Methods:**

Proband and family members were screened for 60 genes involved in X-linked cognitive impairment and the MCT8 mutation was confirmed. Functional consequences of *MCT8* mutations were studied by analysis of [^125^I]TH transport in fibroblasts and transiently transfected JEG3 and COS1 cells, and by subcellular localization of the transporter.

**Results:**

The proband and a male cousin demonstrated clinical findings characteristic of AHDS. Serum analysis showed high T3, low rT3, and normal T4 and TSH levels in the proband. A *MCT8* mutation (c.869C>T; p.S290F) was identified in the proband, his cousin, and several female carriers. Functional analysis of the S290F mutant showed decreased TH transport, metabolism and protein expression in the three cell types, whereas the S290A mutation had no effect. Interestingly, both uptake and efflux of T3 and T4 was impaired in fibroblasts of the proband, compared to his healthy brother. However, no effect of the S290F mutation was observed on TH efflux from COS1 and JEG3 cells. Immunocytochemistry showed plasma membrane localization of wild-type MCT8 and the S290A and S290F mutants in JEG3 cells.

**Conclusions:**

We describe a novel *MCT8* mutation (S290F) in 4 generations of a family with Allan-Herndon-Dudley Syndrome. Functional analysis demonstrates loss-of-function of the MCT8 transporter. Furthermore, our results indicate that the function of the S290F mutant is dependent on cell context. Comparison of the S290F and S290A mutants indicates that it is not the loss of Ser but its substitution with Phe, which leads to S290F dysfunction.

## Introduction

Allan Herndon Dudley Syndrome (AHDS) (OMIM #300523) is a rare X-linked syndrome characterized by cognitive impairment and infantile hypotonia evolving to spastic paraplegia within the first few years of life [1–5]. Other features include low muscle mass with generalized weakness, speech difficulties that range from dysarthria to completely absent speech, variable ataxia and occasional dystonia and/or athetoid movements as well as seizures. Penetrance is complete, although the severity is variable; the most severely individuals affected never achieve ambulation or speech, while the less severely affected (i.e. milder phenotype) may achieve these milestones. All will have significant cognitive impairment.

In 2004, AHDS was associated with loss-of-function mutations in *SLC16A2* (*MCT8*), which codes for monocarboxylate transporter 8 (MCT8), a protein implicated in thyroid hormone (TH) transport [6–8]. From then on, the characteristics of AHDS were extended to include an explicit thyroid profile: high serum T3, low-normal T4, low rT3 and normal-elevated TSH levels. Private mutations have been described in numerous kindreds and singleton cases [9–12]. Currently it is believed that the psychomotor retardation and altered serum TH levels arise from loss of transporter function [13]. Although the exact underlying mechanism that causes the neurologic phenotype remains unclear, it is presumed that subsequent to loss of TH transport, a hypothyroid brain subsists. This hypothyroid state is believed to lead to neurological brain damage as TH is essential during different stages in brain development [14].

This paper describes a novel *MCT8* mutation identified in 4 generations of one family, and correlates the *in vitro* functional findings with the causal role of this mutation in the patients and how it may play a role in the variability of the *in vivo* phenotype.

## Methods

### Patients

The work was done as part of the clinical care for patients with rare conditions. The work was discussed with the Chair of the Children's Hospital of Eastern Ontario REB. REB approval was not required as per institutional REB guidelines. Supporting letter from REB Chair is herewith submitted. For the fibroblasts, verbal consent was obtained and was documented within the patient record. Written consent was obtained for blood collection from the relatives.

The proband and other family members were ascertained through two clinical genetics units. Given the clinical presentation of the proband, a diagnosis of AHDS was suspected. However, other X-linked syndromes remained on the differential. A clinically available next generation sequencing panel for 60 genes involved in X-linked cognitive impairment was performed via Emory Genetics Laboratory (Emory University, Atlanta Georgia, USA), a CLIA (Clinical Laboratory Improvement Amendments) accredited clinical laboratory. PCR amplification of all coding exons contained in the XLID genes was performed on the patient's genomic DNA. Direct sequencing of the amplification products was performed using next generation short base pair read sequencing. The patient's gene sequences were then compared to a normal reference sequence. Sequence variations were classified as mutations, benign variants unrelated to disease, or variations of unknown clinical significance. The assay did not interrogate the promoter region, deep intronic regions, or other regulatory elements, nor assess for large deletions. The mutation was confirmed via Sanger sequencing. Mutation specific testing on other individual family members was performed via amplification of the coding region and flanking intronic sequences of *MCT8* followed by sequencing in the forward and reverse directions. Nucleotide numbering is based on GenBank accession number NM_006517.3.

Chromosomal microarray was performed in 2009 at the Cytogenetics Laboratory of the Hospital for Sick Children (Toronto, Ontario) using the Agilent 44 K custom-designed oligonucleotide EmArray Cyto6000, and analysed using DNA Analytics Version 4.0 (Agilent Technologies, Santa Clara, California, USA).

Thyroid function profiles of the patients and family members were measured in serum for TSH, FT4, T4 and T3 using Vitros ECI technology (Ortho-Clinical Diagnostics, Beerse, Belgium), and for rT3 by radioimmunoassay (Zentech, Angleur, Belgium).

Fibroblasts were obtained from the index patient and his healthy brother.

### Materials

DMEM/F12+glutamax, penicillin/streptomycin, DPBS+Ca^2+^/Mg^2+^, CellMASK, and Alexa Fluor 488 were obtained from Life Technologies (Bleiswijk, The Netherlands [NL]); Vectashield H-1200 from Brunschwig (Amsterdam, NL); culture flasks and dishes from Corning (Schiphol, NL); transfection reagent XtremeGENE 9, FastStart PCR Master, and Protease Inhibitor Cocktail Tablet from Roche (Almere, NL); FBS, BSA, D-glucose, T3, T4, poly-D-lysine, rabbit anti-human (h) MCT8 antibody, poly-D-lysine, paraformaldehyde, and Triton X-100 from Sigma-Aldrich (Zwijndrecht, NL); Precision Plus Protein All Blue Standards from BioRAD (Veenendaal, NL); Tris-HEPES buffer, PAGE-SDS gels from Fisher Scientific (Landsmeer, NL); nitrocellulose membrane from GE Healthcare (Zeist, NL); mouse anti-hGAPDH antibody MAB374 from Chemicon International (Amsterdam, NL); and Na^125^I from Perkin Elmer (Groningen, NL). [^125^I]T3 and [^125^I]T4 were produced in our laboratory as described previously [15].

### Constructs

The pcDNA3-hMCT8 plasmid was obtained as described previously [16]. Mutations were introduced using the QuickChange XL-II Site-Directed Mutagenesis kit (Stratagene, Amstelveen, NL). Primers used for the S290F mutation were: forward 5'-TTCGCCTTTCAGCCATTCCTCGTCATCCTGG-3', and reverse 5'-CCAGGATGACGAGGAATGGCTGAAAGGCGAA-3'; and for the S290A mutation: forward 5' TTCGCCTTTCAGCCAGCCCTCGTCATCCTG 3', and reverse 5' CAGGATGACGAGGGCTGGCTGAAAGGCGAA 3'.

### Cell culture

COS1 and JEG3 cells were obtained from the European Collection of Cell Cultures ECACC (Sigma-Aldrich, Zwijndrecht, NL). Cells were cultured at 37 C and 5% CO2 in culture medium (DMEM/F12+glutamax, 9% FBS, 100 nM Na_2_SeO_3_) plus 1% penicillin/ streptomycin. Fibroblasts were seeded in culture medium in 6-well dishes for transport studies, and in 75 cm^2^ flasks for RNA extraction. COS1 and JEG3 cells intended for transport and metabolism studies were seeded in culture medium in 24-well dishes, and for immunoblotting, RNA isolation and confocal microscopy in 6-well dishes. COS1 and JEG3 cells were transfected using XtremeGENE9 following the manufacturer’s protocol. Experiments were carried out 48 hours after transfection.

### Functional analysis

Experiments were carried out using standard medium (DPBS+Ca^2+^/Mg^2+^/ 0.1% D-glucose) with varying BSA concentrations. Medium volumes are given for 24-well and 6-well dishes, respectively. COS1 and JEG3 cells were transfected in duplicate with 100 ng pcDNA3 (empty vector), wild-type (WT) or mutant pcDNA3-hMCT8. Transport studies were carried out using confluent fibroblasts and transfected cells. To study uptake, cells were rinsed with 0.6–2 ml incubation medium (standard medium + 0.1% BSA), and subsequently incubated for 2–60 minutes at 37 C and 5% CO2 with 1 nM (0.5-1x10^5^cpm) [^125^I]T3 or [^125^I]T4 in 0.5–1.5 ml incubation medium. Some incubations with fibroblasts were prolonged to 24 hours. After incubation, medium was removed and cells were briefly rinsed with 0.6–2 ml incubation medium, lysed in 0.5–1 ml 0.1 M NaOH, and counted in a γ-counter. Protein levels were determined in the lysates using the Bradford protein assay.

To study TH efflux, fibroblasts were preloaded for 60 minutes and transfected COS1 or JEG3 cells for 30 minutes with [^125^I]T3 or [^125^I]T4 as described above. After removing the radioactive medium, cells were directly incubated for 2–30 minutes with 0.5–1.5 ml efflux medium (standard medium with 1%BSA to prevent TH re-uptake). At the indicated times, efflux medium was removed, cells were lysed with 0.1 M NaOH, radioactivity was counted, and protein levels were determined as described above.

To study saturation of T3 uptake by WT and mutant MCT8, experiments were carried out using standard medium without BSA, because T3 binds to BSA in a concentration-dependent manner. Thus, transfected COS1 cells were rinsed with standard medium, and incubated for 10 minutes at 37 C with 1 nM [^125^I]T3 and 0.1–10 μM unlabelled T3 in standard medium. Following incubation, cells were rinsed with standard medium +0.1% BSA, lysed, and further processed as described above.

To study the effects of mutant *vs*. WT MCT8 on intracellular TH availability, T3 and T4 metabolism were studied in COS1 or JEG3 cells expressing the human type 3 deiodinase (D3). Cells were co-transfected with 100 ng pCIneo-hD3 plus 100 ng empty pcDNA3, or WT or mutant pcDNA3-hMCT8 in 24-well dishes. After 48 hours, cells were incubated for 4 hours at 37 C with 1 nM (5x10^5^ cpm) [^125^I]T3 or [^125^I]T4 in incubation medium. TH metabolism was determined by HPLC analysis of the medium as described previously [17].

### mRNA expression of WT and mutant MCT8

COS1 and JEG3 cells were cultured in 6-well dishes and transfected in triplicate with 500 ng pcDNA3, WT or mutant pcDNA3-hMCT8. After 48 hours, RNA was isolated using the High Pure RNA isolation kit (Roche). For RNA extraction from fibroblasts, confluent 75cm^2^ flasks cultured in triplicate were trypsinised, and the cell pellet was processed using the High Pure RNA isolation kit. cDNA was produced from 1 μg RNA using the Taqman Reverse Transcription Reagents kit from Applied Biosystems (Life Technologies). To quantify MCT8 expression levels, 1.25 μl cDNA was added to 11.5 μl mix consisting of FastStart PCR Master and MCT8 primer-probe mix: forward primer 5’-CCATAACTCTGTCGGGATCCTC-3’; reverse primer 5’-ACTCACAATGGGAGAACAGAAGAAG-3’; and probe 5’-FAM-ATACCCATCGCGAGGGCTCCGA-TAMRA-3’. MCT8 mRNA was expressed relative to housekeeping gene *HPRT1* (inventoried assay, Life Technologies).

### Immunoblotting

COS1 and JEG3 cells were transfected in duplicate in 6-well culture dishes with 500 ng pcDNA3, WT or mutant pcDNA3-hMCT8. After 48 hours, cells were rinsed with ice-cold standard medium and incubated for 5 min at 5 C with 200 μl lysis buffer (50 mM HEPES, 150 mM NaCl, 10 mM EDTA, pH 8, 1% Triton X-100, protease inhibitor cocktail). Cell lysates were centrifuged for 15 minutes at 15,000 rpm at 4 C, and treated for 30 minutes at 37 C with 10 mM DTT and 1% SDS. Western blotting was performed as previously described [17]. Briefly, 25 μg supernatant was separated on a 10% SDS-PAGE gel, transferred to nitrocellulose membranes, probed with rabbit anti-hSLC16A2 antibody 1:10,000 and mouse anti-hGAPDH antibody MAB374 1:20,000, and stained with 1:20,000 IRDye 800 CW goat anti-rabbit IgG and 1:20,000 IRDye 680 LT anti-mouse IgG (LI-COR, Westburg, Leusden, NL). Blots were scanned and analysed using the Odyssey 3.0 software.

### Immunocytochemistry

To study subcellular localization of MCT8, JEG3 cells were cultured on poly-D-lysine-coated 24 mm coverslips in 6-well culture dishes. After transfection for 48 hours with 500 ng empty pcDNA3, WT or mutant MCT8, cells were fixed for 5 minutes at 37 C with 4% paraformaldehyde in DPBS, and permeabilised for 5 minutes at room temperature (RT) with 0.2% Triton X-100 in DPBS. After thorough washing, cells were successively incubated (with intermittent washing) for 1 hour at RT with 1:1000 anti-hMCT8 antibody in D-PBS +1% BSA, for 1 hour at RT with 1:1000 2^nd^ antibody Alexa-Fluor 488, and for 5 minutes at 37 C with the plasma membrane marker CellMASK. After washing, coverslips were mounted on glass slides using Vectashield H-1200.

Cells were imaged using a Zeiss LSM510meta confocal laser scanning microscope and a 40x1.3 Plan-Neofluar oil immersion objective. The 405-nm laser line was used together with BP 420–480 emission filter for Hoechst, the 488-nm laser line with BP 505–550 emission filter for MCT8, and the 633-nm laser line with the LP 650 emission filter for CellMASK. Images were deconvolved and corrected for chromatic shifts using Huygens professional software V4.1 (Scientific Volume Imaging, Hilversum, NL). To optimize visualization, contrast was enhanced using Fiji [18].

### Statistical analysis

Results are presented as means ± SEM of 2–3 experiments carried out in duplicate or triplicate. GraphPad Prism 5.01 (GraphPad Software, San Diego California, USA) was used for statistical analysis. A 2-way ANOVA with Bonferroni post-test was used to test differences in uptake and efflux between WT and mutant MCT8, and, if significant, between mutant MCT8 and control (empty vector). To test differences in TH metabolism and MCT8 mRNA expression between control and WT or mutant MCT8 transfected cells, a 1-way ANOVA with Bonferroni post-test was used. A paired t-test was used to test for differences in MCT8 RNA expression in fibroblasts. P<0.05 was considered significant.

## Results

### Clinical Data

**Fig 1:**
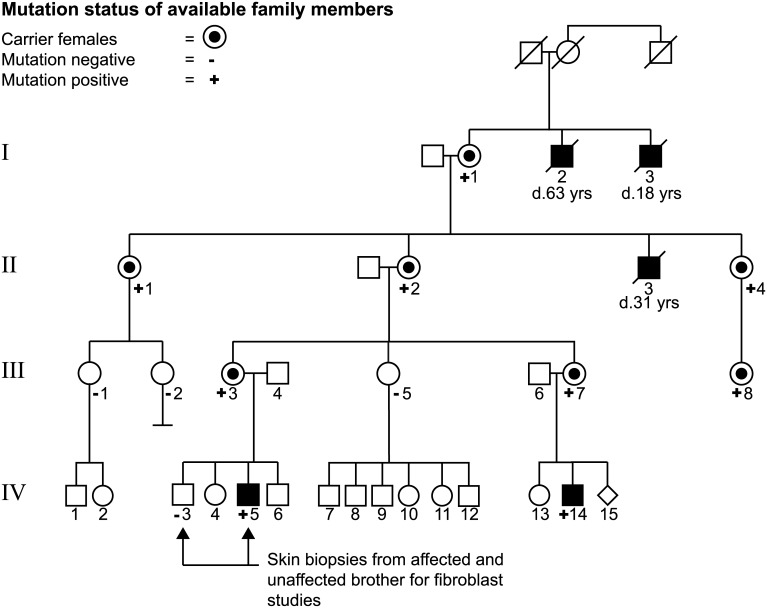
Pedigree of the family with AHDS. The black squares reflect affected males. Circles with a dot represent unaffected heterozygous female carriers. Diamond indicates a pregnancy. Slashed symbols indicate deceased individuals.

The proband and his first-degree cousin presented with early hypotonia and developmental delay. Family history revealed a 4-generation pedigree with 5 affected males in an X-linked pattern of inheritance (Fig 1).

#### Patient A

Patient A, a boy, was initially referred at 4 years for evaluation of profound hypotonia evolving to spasticity, and developmental delays. The pregnancy was associated with reduced fetal movement but prenatal ultrasounds were reported to be normal. He was born at 42 weeks gestation by vaginal vertex delivery after induction of labour with a birth weight of 3.7 kg. His Apgar scores were normal and in the neonatal period, he was slow to regain his birth weight, but there were no specific feeding problems noted.

Developmentally, all aspects were profoundly delayed. He sat independently at 13 months with a prominent C-shaped spine. He first crawled on a four point position at 2 years of age. At 4 years he could take a few steps with assistance, and by 6 years he began ambulating using a walker. At 11 years of age, he was able to walk approximately 500 meters with his walker as well as climb stairs with support. Fine motor skills were also delayed and he was able to feed himself using a spoon at 7 years. He spoke his first words at 3 years and started putting words together at 7 years. At 10 years he was able to speak in full sentences; however, his speech was severely dysarthric. He also required assistance to feed himself, dress, and carry out activities required for personal hygiene. He had truncal instability, required back support to sit, and occasionally had episodes of urinary incontinence. At the last assessment he attended a modified Grade 4 program, was able to use a keyboard, and was making major improvements in picture recognition and in spelling.

There is no history of developmental regression and no episodes of metabolic decompensation. Past medical history is remarkable for febrile seizures on two separate occasions.

At 10 years of age his height was 133 cm (25th centile), weight was 23 kilograms (3rd centile) and head circumference was 52 cm (50th centile). Craniofacial exam revealed deep set eyes, a long face, prominent ear lobes, malar hypoplasia and a highly arched palate. Cardiovascular, respiratory and abdominal examinations were normal. Musculoskeletal examination revealed mild contractures at the hips and knees, and sustained contractures at the ankles bilaterally. He had mild 2,3 syndactyly of the toes and clinodactyly of the 5^th^ fingers. He also had persistent fetal pads of his digits, and bilateral pes planus.

On neurological examination he manifested a significant degree of oromotor dysfunction with constant drooling. The remainder of the cranial nerve examination was otherwise normal. Muscle tone was hypotonic axially but increased peripherally in all four limbs. Muscle bulk was globally reduced. Formal manual muscle strength testing revealed weakness of the deltoids, biceps, and triceps (MRC grade 4/5) but relatively preserved strength of the distal extremities. He had pronounced weakness of the iliopsoas, thigh adductors and abductors, and gluteus maximus as well as quadriceps (MRC grade 4/5). Hamstrings, tibialis anterior were 4/5, gastrocsoleus 4+, and extensor hallucis longus 4; tibialis posterior and peronei could not be tested due to the ankle contractures. Deep tendon reflexes were 2+ bilaterally and plantar response was extensor. Sensation could not be tested.

Cerebellar examination revealed very slow upper extremity movements, but no true dysmetria. He did have an action tremor. Gait analysis revealed a very awkward, unsteady, intoed spastic gait, with his knees bent in a flexed position. Initial contact with the ground was made with his forefoot rather than his heel, with a tendency to toe walk. He was able to take 12 steps without falling.

**Fig 2:**
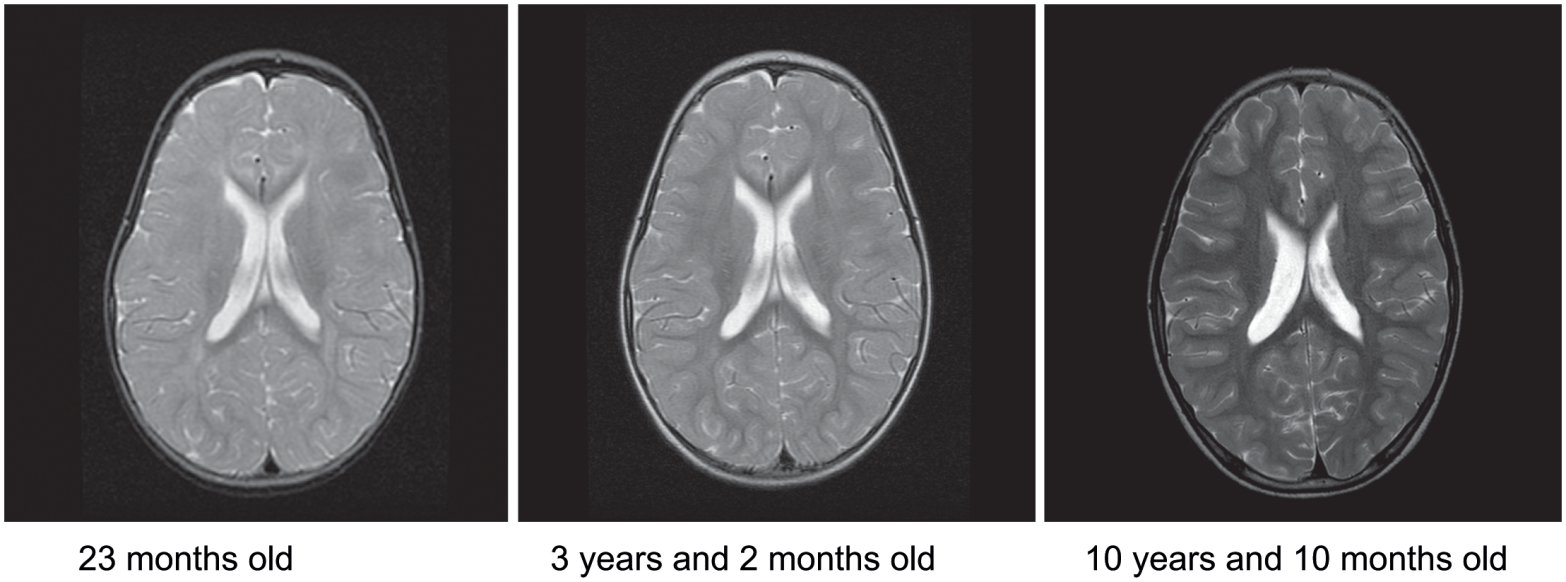
Brain MRI of the index patient. T2-weighted axial images at 23 months, 3 years and 2 months showing delayed myelination, and at 10 years and 10 months myelination has normalized.

MRI of the brain carried out at age 23 months and again at 3 years, were suggestive of delayed myelination (Fig 2) but a repeat study at 10 years was normal. Nerve conduction studies and evoked potentials were normal. Metabolic testing including plasma homocysteine, vitamin B12 level, carnitine profile, very long chain fatty acids, ammonia, plasma amino acids, urine organic acids, urine mucopolysaccharides, lactate, CK, and copper levels were normal. Testing for congenital disorders of glycosylation and disorders of neurotransmitter metabolism was also normal. Karyotype and microarray analyses were normal. Molecular genetic testing for Pelizaeus-Merzbacher Disease, Fragile X, Myotonic Dystrophy, Prader-Willi Syndrome, Spinal Muscular Atrophy and Coffin-Lowry syndrome was normal. A next generation sequencing panel of 60 genes implicated in X-linked cognitive handicap (see methods) revealed a c.C869T mutation of uncertain pathogenicity in *SLC16A2*.

#### Patient B

Patient B is the maternal cousin of patient A, and was also referred for assessment of hypotonia and global developmental delays. He was born at 38 weeks gestation following an uncomplicated pregnancy with a birth weight of 3.6 kg. Prenatal ultrasounds and fetal movements were normal, and there were no concerns in the early neonatal period.

His development was characterized by marked delays in all spheres. He first rolled at 11 months and started commando crawling at 16 months. At 17 months he was not able to sit independently nor was he able to stand. He had no speech but he did babble. His hearing and vision were normal and he is otherwise healthy. At 17 months of age his height was 70 cm (3rd centile), weight was 10.2 kg (3rd centile) and head circumference was 47 cm (25th centile). Craniofacial exam revealed deep set eyes, and mild malar hypoplasia. The ear lobes were up turned and prominent but there were no ear pits or tags. The palate was highly arched but there was no cleft. Respiratory, cardiovascular, abdominal and genitourinary exams were normal. Musculoskeletal exam revealed deep palmar creases, as well as tapering of his fingers. Examination of the feet revealed bilateral 4th and 5th toe clinodactyly. He also had joint laxity of upper and lower extremities.

On neurological exam he had evidence of poor oromotor control and drooled constantly. Muscle tone was globally hypotonic, both axially and peripherally, but muscle strength was well preserved. Deep tendon reflexes were 2+ bilaterally and plantar response was extensor. Formal cerebellar and sensory testing could not be carried out.

An MRI of the brain at 9 months revealed delayed myelination as well as thinning of the corpus callosum. CK, plasma amino acids, urine organic acids, and karyotype were normal. Molecular analysis of *MCT8* revealed the same c.C869T mutation as patient A.

### Family History

Patient A (IV.4) is the third child of non-consanguineous parents of Northern European descent (Fig 1). His 10 year old brother (IV.3), 9 year old sister (IV.4) and 5 year old brother (IV.5) are healthy. His sister does have learning difficulties and has a modified program. A maternal aunt (III.7, mother of patient B) has atrioventricular nodal re-entry tachycardia but is otherwise healthy. The maternal grandmother (II.2) is healthy and has two sisters (II.1,4) with scoliosis, and one is reported to have mild cognitive delays. Female relatives were not formally assessed in regard to cognitive function. A brother (II.3) was profoundly cognitively impaired, died at 35 years of age, and was never able to talk or walk independently. According to the family, his clinical symptoms were similar to those of patients A and B. The maternal great-grandmother (I.1) is alive and well. There were two maternal great-granduncles (I.2,3), both of whom were known to have significant cognitive impairment, hypotonia that evolved to spasticity and were never able to live independently. One of these great-granduncles was able to walk with support and had some dysarthric speech. Patient A's father (III.4) had seizures in childhood, as did his twin brother. There is a paternal family history of seizures.

Patient B (IV.14) has one 5 year old sister (IV.13) who is healthy. His father (III.6) is healthy with no cognitive or neurological issues. There is a paternal great aunt who had a child with Down syndrome. There is no history of consanguinity, other history of cognitive impairment or developmental delay, recurrent pregnancy loss or other neurological conditions.

### Serum thyroid parameters

**Fig 3:**
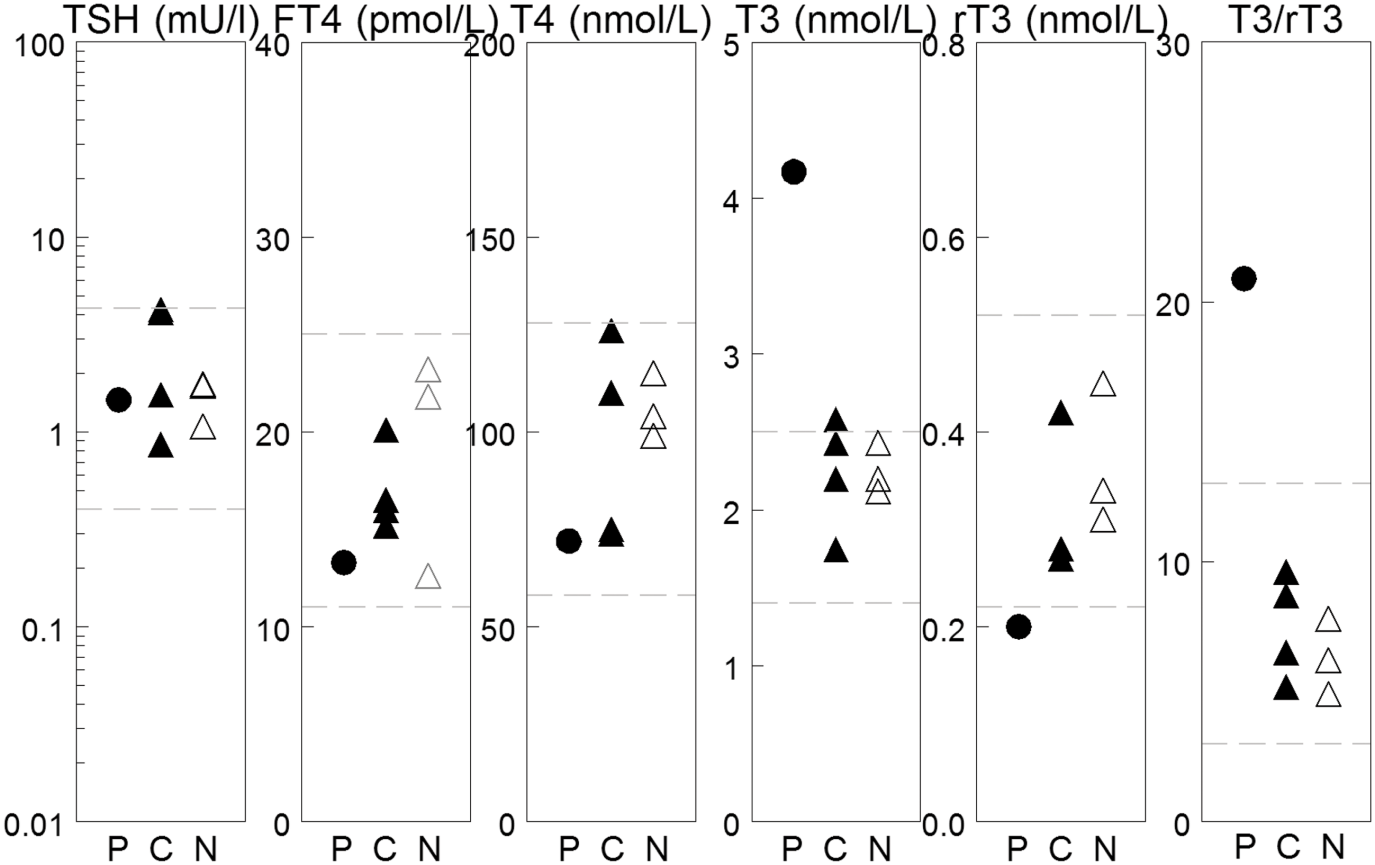
Thyroid hormone serum levels in proband and family. The dashed lines reflect the boundaries of the normal range for adults of the respective test. P = patient, C = carrier and N = unaffected family members.

One of the key findings in MCT8 patients is their markedly elevated serum T3 levels combined with low FT4 and normal to elevated TSH levels. Therefore we measured serum thyroid parameters in the proband, carriers and non-carriers in this family. The proband has a FT4 and TSH within the normal range; a markedly increased T3 of 4.17 nmol/l and a slightly decreased rT3 of 0.20 nmol/l (Fig 3). Serum T3 was at the upper limit of normal in different relatives irrespective of carrier status. Serum (F)T4, rT3 and TSH were normal in all relatives tested. Serum from patient B and other relatives were not available for analyses.

### Functional analysis

**Fig 4:**
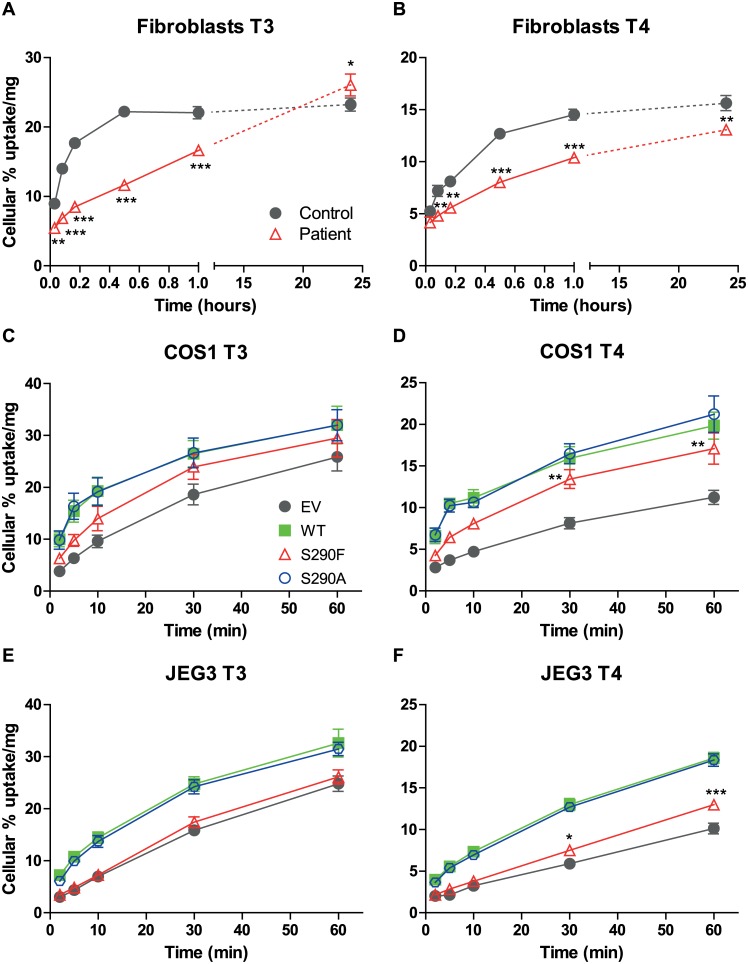
Cellular uptake of [^125^I]T3 and [^125^I]T4 by WT and mutated MCT8. Time dependent cellular uptake of [^125^I]T3 (A,C,E) and [^125^I]T4 (B,D,F) in fibroblasts (A,B), and in transiently transfected COS1 cells (C,D) and JEG3 cells (E,F). Uptake is shown as percentage of added T3 or T4 and corrected for protein. Results are presented as mean ± SEM (n = 3). Significance is indicated for control *vs*. patient fibroblasts (A,B). C-F Mutual difference of WT MCT8 and mutants was tested; if significant, difference with control (EV) was tested. *P <0.05; **P <0.01; ***P <0.001.

To explore the functional consequences of the MCT8 mutation, TH transport studies were performed in fibroblasts from the index patient and his healthy brother. Fig 4A and 4B demonstrate that [^125^I]T3 and [^125^I]T4 uptake were significantly impaired in the index patient compared to his healthy brother. However, uptake of T3 and T4 by the control fibroblasts appeared to approach a plateau after 1 hour, whereas it continued to increase in the patient’s fibroblasts. After 24 hours, cellular [^125^I]T3 was significantly higher (26% *vs*. 23%) but cellular [^125^I]T4 was still lower (13% *vs*.16%) in patient *vs*. control fibroblasts. These results suggest that not only uptake but also efflux of T4 and, in particular, T3 are impaired by the MCT8 mutation.

The functional characteristics of the mutation were investigated in more detail in COS1 and JEG3 cells transiently transfected with WT or mutant MCT8. Cells transfected with empty vector served as controls. In addition to the S290F mutation identified in the family, we also studied the S290A mutation to gain insight into the importance of the Ser residue. Ser possesses a functional OH group, deletion of which results in Ala; its substitution with a phenyl group in Phe is expected to have a much greater impact on protein structure. After correcting for background uptake in control cells, uptake after 30 minutes of both T3 and T4 by the S290F mutant in COS1 cells was decreased by 32% compared with WT MCT8 (Fig 4C and 4D). The S290A mutant showed identical uptake of T3 and T4 in COS1 cells as WT MCT8. In contrast, T3 and T4 uptake by the S290F mutant was completely abolished in JEG3 cells (Fig 4E and 4F). Only after prolonged incubation for 30 and 60 minutes, a significant 1.3-fold increase in T4 uptake by the S290F mutant was observed compared to control JEG3 cells. Similar to the results obtained in COS1 cells, uptake of T3 and T4 was identical between the S290A mutant and WT MCT8.

**Fig 5:**
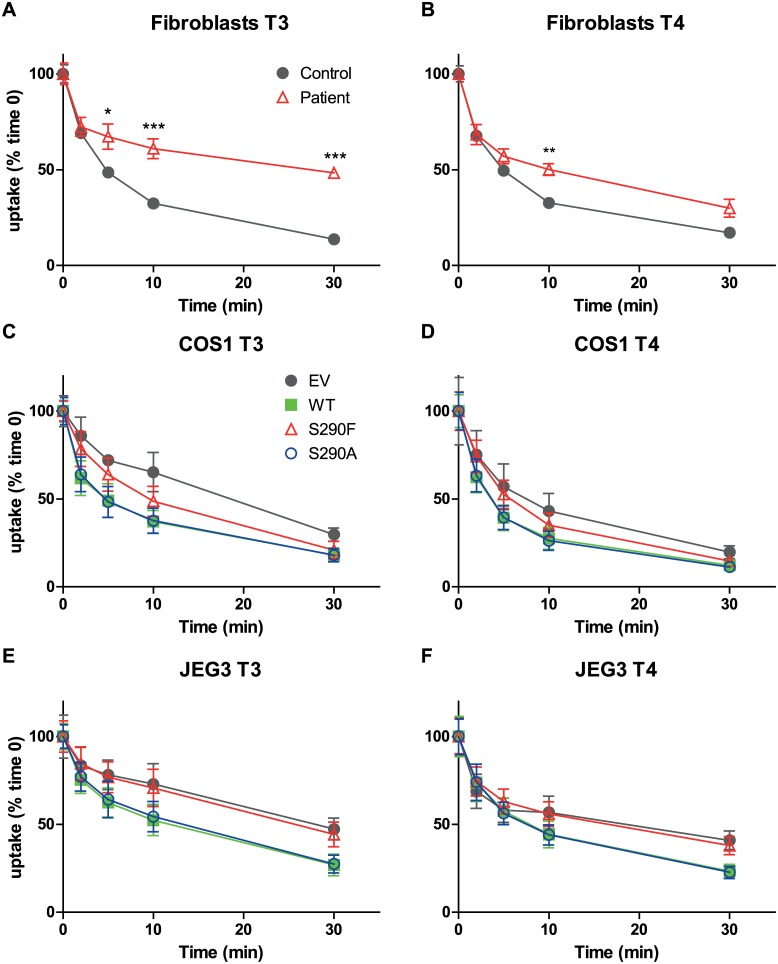
Efflux of [^125^I]T3 and [^125^I]T4 by WT and mutated MCT8. Efflux of [^125^I]T3 (A,C,E) and [^125^I]T4 (B,D,F) for 2–30 min from fibroblasts (A,B), and transiently transfected COS1 cells (C,D) and JEG3 cells (E,F). Efflux is shown as percentage of cellular radioactivity at 0 min and is corrected for protein. Results are presented as mean ± SEM (n = 3). Significance is indicated for control *vs*. patient fibroblast (A,B) or mutual difference between WT MCT8 and mutants. *P <0.05; **P <0.01; ***P <0.001.

Because the fibroblast uptake studies suggested impaired T3 and T4 efflux from the patient’s cells, we decided to study [^125^I]T3 and [^125^I]T4 efflux directly from patient’s and control fibroblasts. Fig 5A shows a significantly impaired T3 efflux from patient’s fibroblasts. T4 efflux was also reduced but not to the same degree as T3 efflux (Fig 5B). Only at 10 minutes a significant difference was found with T4 efflux from control fibroblasts. In contrast, T3 and T4 efflux were not significantly different between COS1 or JEG3 cells transiently transfected with WT MCT or the S290F mutant (Fig 5C–5F). The S290A mutant was not significantly different from WT MCT8 in mediating T3 and T4 efflux from both cell types.

**Fig 6:**
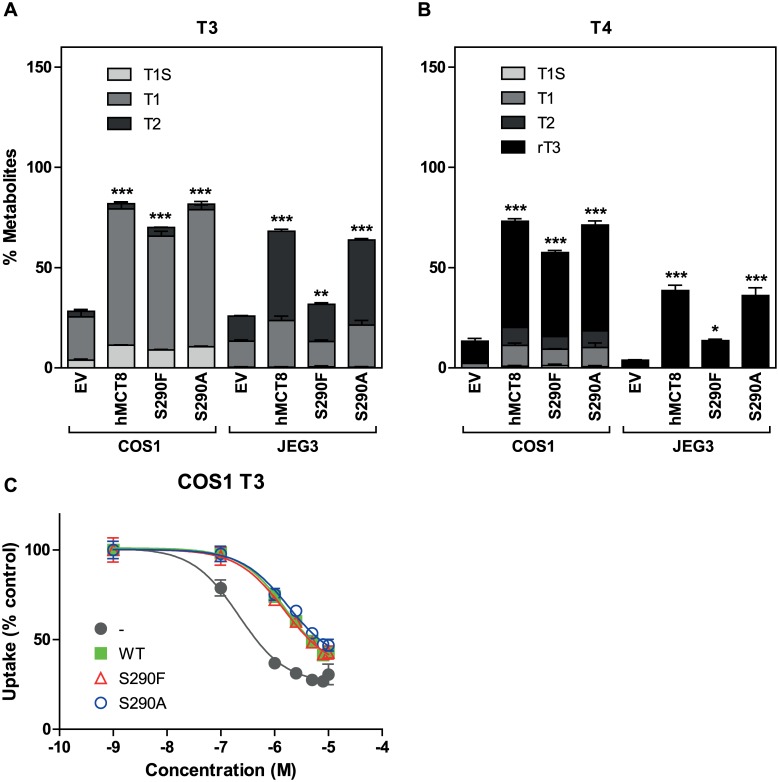
Metabolism of [^125^I]T3 and [^125^I]T4 by D3 and T3 uptake saturation in MCT8 expressing cells. Metabolism of [^125^I]T3 (A) and [^125^I]T4 (B) after 4 hours in COS1 and JEG3 cells co-transfected with pCIneo-hD3 and empty vector, WT or mutant MCT8. Metabolism is shown as % of radioactivity in the incubation medium. **C** Inhibition of [^125^I]T3 uptake by WT and mutated MCT8 by increasing concentrations of unlabelled T3. Results are presented as mean ± SEM (n = 3). Significance is indicated for D3 co-transfected with WT or mutant MCT8 *vs*. D3 alone. *P <0.05; **P <0.01; ***P <0.001.

To study the effects of the S290F and S290A mutations on the intracellular TH availability, we studied the metabolism of [^125^I]T3 and [^125^I]T4 in JEG3 and COS1 cells co-transfected with D3 and WT or mutant MCT8. Stimulation of intracellular deiodination by WT MCT8 was 2.9-fold for T3 and 8.1-fold for T4 in COS1 cells, and was 2.6-fold for T3 and 10.1-fold for T4 in JEG3 cells (Fig 6A and 6B). Relative to WT MCT8, the activity of the S290F mutant in facilitating intracellular deiodination was 85% for T3 and 74% for T4 in COS1 cells, and 47% for T3 and 35% for T4 in JEG3 cells. These findings indicate that the S290F mutant has relatively high activity in stimulating intracellular TH availability in COS1 cells, although low but significant facilitation of T3 and in particular T4 deiodination was also observed in JEG3 cells. No difference in facilitating intracellular TH metabolism was observed between WT MCT8 and the S290A mutant.

To further characterise differences in transport activity between WT MCT8 and the mutants, we determined apparent affinities of T3 transport in transfected COS1 cells. This was done by studying the progressive inhibition of [^125^I]T3 uptake by increasing concentrations of unlabelled T3. Fig 6C shows that the inhibition curves for WT MCT8, and both mutants largely overlapped, and were shifted to higher T3 concentrations compared with control COS1 cells. IC_50_ values amounted to 1.7 μM for WT MCT, 1.5 μM for the S290F mutant and 1.8 μM for the S290A mutant, compared with 0.22 μM for control cells. These findings suggest similar affinities of T3 transport in COS1 cells transfected with WT MCT8 or either mutant. These findings confirm that MCT8 has relatively high Km values, in particular in comparison with serum TH levels. Therefore, the rate of TH uptake by MCT8 is not limiting at even thyrotoxic hormone levels but will be determined by the level of MCT8 expression.

### Expression and cellular distribution

**Fig 7:**
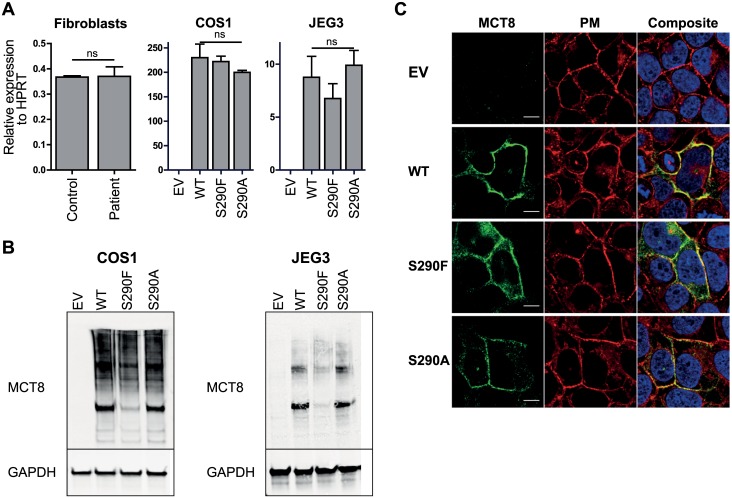
RNA (A), protein expression (B) and cellular distribution (C) of WT and mutant MCT8. **A** MCT8 mRNA levels relative to housekeeping gene HPRT1 in fibroblasts, and transiently transfected COS1 and JEG3 cells. **B** Western blots of lysates of transiently transfected COS1 and JEG3 cells. The blots show a specific MCT8 monomer band of 60 kDa and homodimer band of 120 kDa. GAPDH was used as a housekeeping protein. **C** Confocal imaging of transiently transfected and probed JEG3 cells. The cellular distribution of MCT8 proteins is shown in green, nuclear staining with Hoechst in blue, and plasma membrane (PM) staining with CellMASK in red. The yellow signal in the composite image reflects co-localization of the PM marker and MCT8, indicating that MCT8 is expressed at the PM. Images were deconvolved and corrected for chromatic shift using Huygens. Contrast was enhanced to optimize visualization. Scale bar represents 10 μm.

Loss-of-function mutations may be caused by different mechanisms at the RNA or protein level. Therefore, we determined MCT8 mRNA levels in fibroblasts and transiently transfected COS1 and JEG3 cells relative to the housekeeping gene *HPRT1* (Fig 7A). No significant difference was found between fibroblasts from patient A and his healthy brother nor between COS1 or JEG3 cells transfected with WT MCT8 or the S290F or S290A mutant.

Subsequently, we studied protein expression levels in transiently transfected COS1 and JEG3 cells by immunoblotting. In both cell lines, WT MCT8 and both mutant proteins were present as ~60 kDa monomer and ~120 kDa homodimer (Fig 7B). In both cell lines, the S290F protein was expressed at a markedly lower level than WT MCT8 and the S290A mutant. Unfortunately, expression of MCT8 protein in fibroblasts is too low to be detected by immunoblotting and immunocytochemistry [19].

Finally, we assessed the cellular distribution of WT and mutant MCT8 using immunocytochemistry in transiently transfected JEG3 cells. We did not carry out these experiments in COS1 cells because of difficulties in obtaining suitable plasma membrane staining using CellMASK [17]. Fig 7C shows the cellular distribution of WT MCT8 and both mutants. No fluorescent signal was present in control cells, supporting the specificity of the MCT8 antibody. The composite images show a clear co-localization of the plasma membrane marker with WT MCT8 as well as with both S290A and S290F mutants, although the number of cells with substantial expression of in particular the S290F mutant was limited. Nonetheless, the S290F mutant appears to reach the plasma membrane.

## Discussion

We present the clinical and molecular characteristics of the MCT8 S290F mutation in a 4-generation family with AHDS, which segregates with the phenotype and obligate carrier status within the family. Additionally, we demonstrate that the function of the S290F mutation is dependent on the cellular context, and that it is not the loss of Ser but its substitution with Phe, which affects MCT8 function.

Functional analysis demonstrated that the S290F mutation produces a significant loss of MCT8 function in fibroblasts of the index patient and in transiently transfected JEG3 cells, but that results in transfected COS1 cells are less clear-cut. Similar discrepancies in the effects of MCT8 mutations on TH transport and metabolism in COS1 and JEG3 cells have been observed before [17]. Interestingly, not only uptake but also efflux of T4 and T3 was impaired in the patient’s fibroblasts; but no effect of the S290F mutation was seen on TH efflux from COS1 and JEG3 cells. These findings suggest that the function of this MCT8 mutant depends on the cellular context and implies that a MCT8 mutation may have different consequences in different tissues. The cell-type dependent residual activities of MCT8 mutants supports our hypothesis that the loss of MCT8 function depends on other interacting proteins [17].

Loss-of-function mutations in MCT8 could involve different mechanisms, *i*.*e*. impaired synthesis, disturbed protein folding, defective trafficking, increased protein degradation, and loss of TH binding and transport [20]. Given the similar MCT8 mRNA levels in fibroblasts of the patient and his unaffected brother as well as in cells transiently transfected with WT or mutant MCT8, impaired synthesis is unlikely to be an important pathogenic mechanism. In transiently transfected COS1 and JEG3 cells, levels of the mutant S290F protein were substantially lower than the WT and S290A proteins. Immunocytochemical analysis of transfected JEG3 cells demonstrated that the S290F protein undergoes proper trafficking to the plasma membrane although in lower amounts than the WT and S290A proteins. The apparent affinity of T3 for the S290F mutant expressed in COS1 cells appears to be unaffected. Taken together, these findings suggest that lowered protein expression is a major mechanism for the loss of MCT8 function caused by the S290F mutation, which may be due to decreased protein synthesis and/or increased protein degradation, although no degradation products were visible on the immunoblots.

As mentioned in the methods section, mutation of Ser to Ala is unlikely to affect protein folding in contrast to the substitution with the much larger Phe residue. The S290A mutation does not affect MCT8 function which suggests that the Ser residue is not essential for TH transport. The results rather indicate that it is the substitution with Phe that causes the loss of MCT8 function. This substitution may disturb MCT8 folding with cell type-dependent consequences for TH transport, possibly related to differential expression of MCT8-interacting proteins.

Clinical correlation can be attempted from the functional results herein. Several mutations have been demonstrated to confer a milder phenotype [19–21]. Although the affected individuals described vary in their presentations, the phenotype in this family generally appears to be a milder one given that the proband and one of his great-granduncles gained the ability to walk and speak, albeit with difficulty. The younger affected cousin is currently learning to walk. While other factors could certainly play a role in this milder phenotype, such as increased intervention in the form of physiotherapy or other genetic modifiers, the data suggests the mutation does not completely abrogate transporter function. Specifically, the S290F mutation in MCT8 causes intracellular TH deficiency, but because of minimal uptake and impaired efflux, some TH activity remains to produce a milder phenotype.

As previously described, our functional studies in transfected cells have some limitations as they represent overexpression models [17]. On the other hand, by using different cell types for transfection it has become more and more evident that the effects of MCT8 mutations depend on the cellular context [17, 22]. MCT8 is expressed in variety of tissues, including brain, liver, kidney and skeletal muscle, and in different cell types, *e*.*g*. in endothelial cells, choroid plexus epithelial cells, neurons, oligodendrocytes, and tanycytes in brain [23–27]. Therefore, the *in vivo* effects of *MCT8* mutations may vary greatly between these tissues and cell types. Use of different cell types for functional analysis adds to our understanding of the tissue-specific effects of *MCT8* mutations and provides insights to unravel the underlying pathogenic mechanism.

AHDS may represent an under-recognized etiology for X-linked cognitive impairment particularly when presenting as singleton cases (*e*.*g*. no family history of similar) and because of the clinical overlap with the much commoner cerebral palsy [10]. Greater understanding of MCT8 transporter function and mutations is important; for allowing accurate diagnosis of other uncharacterized mutations, for providing information toward prognosis, and for informing the basis for future therapy. Promise for therapy exists in the form of TH analogs such as DITPA, Triac and Tetrac, thyromimetics not dependent on MCT8 transporter activity [28–30]. In theory, these analogs bypass MCT8 and access TH targets within cells to modify TH-dependent genes. Though the first thyromimetic (DITPA) study in MCT8 patients did not improve cognitive development, it improved peripheral thyrotoxicosis [28]. Studies are ongoing to test the possible beneficial effects of other thyromimetics. As brain development continues after birth [14], this is of particular interest for families with milder presentations, such as the family described here, since early therapy that may even only slightly increase TH activity could have significant clinical implications.

In summary, our findings suggest that the S290F mutation in MCT8 identified in this AHDS family results in decreased MCT8 protein expression with consequently impaired T3 and T4 transport. The residual uptake combined with the impaired efflux may permit sufficient intracellular TH to produce a milder phenotype in this family compared with most other reported patients with AHDS. The cell type-dependent effects of the S290A mutation on MCT8 function observed *in vitro* suggest that *MCT8* mutations may also affect TH transport in patients in a tissue-dependent manner.
